# AaMYB3 interacts with AabHLH1 to regulate proanthocyanidin accumulation in *Anthurium andraeanum* (Hort.)—another strategy to modulate pigmentation

**DOI:** 10.1038/s41438-018-0102-6

**Published:** 2019-01-01

**Authors:** Chonghui Li, Jian Qiu, Surong Huang, Junmei Yin, Guangsui Yang

**Affiliations:** 1 Tropical Crops Genetic Resources Institute, the Chinese Academy of Tropical Agricultural Sciences (CATAS) / Key Laboratory of Crop Gene Resources and Germplasm Enhancement in Southern China, Ministry of Agriculture, Danzhou, 571737 China; 2 The Engineering Technology Research Center of Tropical Ornamental Plant Germplasm Innovation and Utilization, Hainan Province, Danzhou, 571737 China; 3 Rubber Research Institute, CATAS/ Key Laboratory of Biology and Genetic Resources of Rubber Tree, Ministry of Agriculture, Danzhou, 571737 China

**Keywords:** Plant molecular biology, Plant breeding

## Abstract

Proanthocyanidins (PAs), also known as “condensed tannins”, are colorless metabolites produced through the flavonoid pathway that are involved in stress resistance in plants. Because PAs are involved in the anthocyanin biosynthetic pathway, they play a role in the modification of pigmentation conferred by anthocyanins in ornamental organs. In this study, we isolated the gene and functionally characterized an R2R3-MYB transcription factor (TF), AaMYB3, and a basic helix-loop-helix TF, AabHLH1, from *Anthurium andraeanum* (Hort.), a typical tropical flower. *AaMYB3* is primarily expressed in the spathe and negatively correlates with anthocyanin accumulation. A complementation test in an *Arabidopsis tt8* mutant showed that AabHLH1 successfully restores the PA-deficient seed coat phenotype. The ectopic overexpression of *AaMYB3* alone or its coexpression with *AabHLH*1 in transgenic tobacco resulted in light pink or even pale-pink corolla limbs by reducing their anthocyanin levels and greatly enhancing their accumulation of PAs. This overexpression of the anthurium TF genes upregulated the late anthocyanin enzyme-encoding genes (*NtDFR* and *NtANS*) and the key PA genes (*NtLAR* and *NtANR*) in transgenic tobacco. The interaction between AaMYB3 and the AabHLH1 protein was confirmed using yeast two-hybrid (Y2H) and bimolecular fluorescence complementation (BiFC) assays. In the developing red spathes of the cultivars “Vitara” and “Tropical”, the expression of *AaMYB3* was closely linked to PA accumulation, and *AaMYB3* was coexpressed with *AaCHS*, *AaF3H*, *AaDFR*, *AaANS*, *AaLAR*, and *AaANR*. The expression pattern of *AabHLH1* was similar to that of *AaF3′H*. Our results suggest that AaMYB3 and AabHLH1 are involved in the regulation of PA biosynthesis in anthurium and could potentially be used to metabolically engineer PA biosynthesis in plants.

## Plant physiology: Regulating the biosynthesis of survival mechanisms

Two genes have been discovered that drive the production of survival-associated molecules in plants. Proanthocyanidins (PAs) are anti-inflammatory and antioxidant molecules produced by plants that defend against pathogens and preditors. A Chinese research team led by Junmei Yin and Guangsui Yang, from the Chinese Academy of Tropical Agricultural Sciences, set out to discover the genes responsible for PA biosynthesis, finding that two proteins, AaMYB3 and AabHLH1, interact and contribute to PA biosynthesis in the tropical flower *Anthurium andraeanum*. Their results also showed that genetically overexpressing the two proteins caused PA accumulation in tobacco plants, with the function of AaMYB3 increased by AabHLH1. The expression pattern of the *AaMYB3* and *AabHLH1* genes coincided with other PA biosynthetic genes. The researchers suggest these genes may be utilized to engineer PA biosynthesis in plants.

## Introduction

Proanthocyanidins (PAs), the end products of the flavonoid biosynthetic pathway, occur in the fruits, bark, leaves, and seeds of many plants^1^. PAs have strong antioxidant properties, and their primary function in plants is to defend against pathogens, insects, diseases, and larger herbivores^1,2^. PAs, also called “condensed tannins”, confer astringency upon plants that originally served as forage and function as herbivore feeding deterrents^1,3^. The dietary PAs in wine, fruit juices, teas, and cocoa contribute to their taste and health benefits through their antioxidant and radical-scavenging functions and their anti-inflammatory activities^4^. Therefore, there is strong interest in the molecular biosynthesis and metabolic engineering of PAs in crops and fruits^5,6^.

PA biosynthesis shares the same upstream pathway with anthocyanins, although they are subsequently synthesized as the oligomeric or polymeric end products of one of several branches of the flavonoid pathway in the final catalytic steps^4^. The formation of the flavan-3-ols begins with the dihydroflavanol 4-reductase (DFR)-mediated reduction of dihydroflavonols to leucoanthocyanidins, which are then reduced by leucoanthocyanidin reductase (LAR) to catechin. The other way that flavan-3-ols (epicatechin) are formed by the reduction of anthocyanidin, which is converted from leucoanthocyanidins by anthocyanidin synthase (ANS), which is catalyzed by anthocyanidin reductase (ANR). Flavonoid biosynthesis is primarily controlled by transcription factors (TFs) that regulate the expression of the genes encoding the biosynthetic enzymes in the associated pathways. In most plants, the process is conservatively regulated by the MBW protein complex formed by the combination of R2R3-MYB, basic helix-loop-helix protein (bHLH) and the WD40 repeat-containing protein (WDR)^7^. The regulation of PA biosynthesis by the MBW complex has been well characterized in *Arabidopsis* with the TT2 (MYB)–TT8 (bHLH)–TTG1 (WDR) model^8^. The ternary transcription protein activates the late anthocyanin and PA-specific genes, including *DFR*, *ANS*, and *BAN* (also known as *ANR*)^8–10^. The homologs of TT2, TT8, and TTG1 in other plants have since been discovered. For example, in strawberry, FaMYB9/FaMYB11, FabHLH3, and FaTTG1 form a complex that upregulates the expression of *ANS* and *LAR*, therefore increasing the levels of PAs^11^. In persimmon, DkMYB2 and DkMYB4 interact with both DkMYC1 (bHLH) and DkWDR1 to regulate PA accumulation in the fruit^12^. The WDR proteins in the MBW complex are thought to confer a docking platform for the MYB–bHLH interaction. Many studies have suggested that R2R3-MYB is a key factor that determines the activity that induces or represses the transcription of the PA biosynthetic genes. For example, in grapevine, VvMYBPA1^13^, VvMYBPA2^14^, and VvMYBPAR^15^ promote PA accumulation, and VvMYBC2-L1^16^ negatively regulates PA accumulation by downregulating the expression of the PA genes. Therefore, much attention has been paid to the role of the MYB TF in the PA transcriptional regulation process. Many MYBs have been identified as PA regulators, such as poplar PtMYB134^17^, *Trifolium arvense* TaMYB14^18^, apple MdMYB9^19^, peach PpMYB7^20^, and even in the ornamental plants coleus (SsMYB3^21^) and *Malus* crabapple (MdMYB12b^22^). Many of these genes are considered suitable candidates to metabolically engineer PA biosynthesis in plants, because MYBs regulate multiple key enzyme-encoding genes, and therefore have advantages over single key enzymes in such gene-based strategies^21^.

*Anthurium andraeanum* (Hort.) is a well-known tropical flower with a colorful spathe and spadix and is produced commercially as a cut flower or potted plant^23^. Anthurium has a long horticultural history and is very important in worldwide trade. The color of the spathe and spadix is one of the most important traits of anthuriums. The anthocyanins cyanidin, pelargonidin and peonidin, in the form of anthocyanidin 3-rutinoside, are primarily responsible for the red, purple, pink, orange, and coral coloration of the anthuriums^24^. The biosynthetic pathways and the expression pattern of the key enzyme genes in anthocyanin biosynthesis have been characterized in anthurium^23,25,26^. However, in the MBW complex, only the MYB genes have so far been identified in anthurium by their ectopic expression. AaMYB1^27^ is involved in the positive regulation of anthocyanin biosynthesis, and AnAN2 may act as a negative regulator of anthocyanin production^28^. We previously isolated the R2R3-MYB gene *AaMYB2* and demonstrated that *AaMYB2* expression is closely related to anthocyanin accumulation and that AaMYB2 primarily contributes to the regulation of anthocyanin biosynthesis in the anthurium spathes and leaves^29^. However, the regulatory mechanism of the biosynthesis of flavonoids, including anthocyanins and PAs, in anthurium is still unclear.

PAs are colorless flavonoid polymers that are later pathway products downstream from anthocyanins. The ectopic expression of *ANR* in *Nicotiana tabacum* (tobacco) flowers and *Arabidopsis* leaves resulted in the loss of anthocyanins and the accumulation of PAs, suggesting that the excessive accumulation of PAs causes a reduction in anthocyanin^30,31^. Similarly, the expression pattern of *ANR* correlates negatively with anthocyanin accumulation in the anthurium spathe^32^. Therefore, PA accumulation in these ornamental plant organs dilutes the pigmentation contributed by anthocyanins, thus playing an important role in coloration. However, very few studies have reported the key structural and regulatory genes involved in the PA biosynthetic pathway in ornamental plants. Therefore, the isolation and characterization of the genes that regulate this pathway are necessary to understand the molecular regulation of PA and anthocyanin biosynthesis in this species^21^.

In this study, we present the isolation of the first gene encoding a bHLH protein and one gene encoding an R2R3-MYB TF in anthurium, designated AabHLH1 and AaMYB3, respectively. A phylogenetic analysis indicated that AaMYB3 and AabHLH1 are homologous to the *Arabidopsis* PA regulators AtTT2 and AtTT8, respectively. The functions of AaMYB3 and AabHLH1 were demonstrated with a complementation test in an *Arabidopsis* mutant and their exogenous expression in tobacco. Our results demonstrate that AaMYB3 interacts with AabHLH1 and that they are involved in PA biosynthesis in anthurium. Based on these results and our previous study, we propose a model of the regulation of anthocyanin and PA accumulation in the anthurium spathe, which extends our understanding of the whole flavonoid metabolic pathway in anthurium. These two TFs, AaMYB3 and AabHLH1, may also be useful in the metabolic engineering of PA biosynthesis in plants.

## Materials and methods

### Plant materials

Mature 6-year-old plants of seven *A. andraeanum* (Hort.) cultivars maintained in a shade greenhouse at the Tropical Flower Resource Garden, Tropical Crops Genetic Resources Institute, Chinese Academy of Tropical Agricultural Sciences (Danzhou, Hainan province, China) were used in this study. The cultivars were “Tropical” and “Vitara” (red-spathed), “Pink Champion” (pink), “Cheers” (light pink), “Rapido” (purple), “Acropolis” (white), and “Midori” (green). Floral tissue samples, including the spathe and the spadix, were collected between 9 A.M. and 10 A.M. in November 2016 from the developmental stages 1 to 5 of the spathe as described by Li et al.^29^. They were stage 1, the flower (including the spathe and spadix) fully protruded from the protection sheath; stage 2, the floral peduncle elongated but the spathe tightly furled; stage 3, the spathe was half unfurled; stage 4, the spathe was newly fully expanded, and stage 5, the color at the lower two-thirds of spadix became shallow. Simultaneously, the newly fully expanded brown leaves and green mature leaves and the peduncle of flower at stage 4 of “Tropical” were also obtained. Three biological replicates (three distinct spathes or spadix or leaves) within five random plants were selected and used for the subsequent metabolite and RNA analyses.

*Nicotiana tabacum* cv. Wisconsin 38 was used in the gene overexpression experiments. All the tobacco plants were grown in a 50% shade greenhouse under natural sunlight. The corolla limbs of the T_1_ transgenic tobacco plants were collected between 9 A.M. and 10 A.M. in April 2017. Three biological replicates (three distinct tobacco flowers) within five plants were selected and used for the subsequent metabolite and RNA analyses.

*Arabidopsis thaliana* ecotype “Columbia” was used as the wild type control. In addition, the *tt2* and *tt8* Arabidopsis mutants (SALK 005260 and SALK 063334, respectively) obtained from the Arabidopsis Biological Resource Center, were used as the background for the genetic transformation and the negative control. All the *Arabidopsis* plants were grown in 14-h days and 10-h nights at 23°C in a growth chamber.

### Anthocyanin, flavonoid, and polyphenol contents measurement

The extraction and the content measurement of the anthocyanins were performed as described by Li et al.^29^. The total flavonoids and polyphenols were extracted and measured as described previously^33^ with slight modifications. The extracts were prepared using fresh tissue samples extracted with methanol. The experiment was repeated three times for each sample.

### PA extraction and determination

The dimethylaminocinnamaldehyde (DMACA) stain method was used to evaluate the PA content. Soluble PAs from fresh anthurium tissues and tobacco corolla limbs were extracted and measured as described previously^34^. Epicatechin was used as a standard for PA quantification^35^. The experiment was repeated three times for each sample.

### RNA isolation and cDNA synthesis

The total RNA was extracted from anthurium tissues using an RNAprep Pure Plant Kit (Polysaccharides & Polyphenolics-rich) (TIANGEN, Beijing, China). The total RNA from the *Arabidopsis* leaves and tobacco corolla limbs was extracted using a Plant Total RNA Isolation Kit (FOREGENE, Chengdu, China). The cDNA was synthesized according to the manufacturer’s instructions for the RevertAid First Strand cDNA Synthesis Kit (Thermo Scientific, Waltham, MA, USA).

### Isolation of the full-length cDNA of *AaMYB3* and *AabHLH1* and their sequence analysis

One MYB unigene and one bHLH unigene were selected from the mixed floral and foliar transcriptome database of anthurium^29^, which were annotated as AtTT2-like and AtTT8-like transcription factors, respectively. The unigenes were designated as *AaMYB3* (GenBank accession no. MH349476) and *AabHLH1* (accession no. MH349477). The full-length cDNA of *AaMYB3* and *AabHLH1* was isolated from the “Tropical” spathe with reverse transcription (RT)-PCR using primers designed according to the transcriptome data: forward 5′-ATGGGCAGGAGACCCTGTT-3′, reverse 5′-CGCCATTACTTCACCCATTC-3′ for *AaMYB3*, and forward 5′-AGGAGGGGTAGTTGAGCAGGT-3′, reverse 5′-TCATGCTCTAAGCATGTCACGA-3′ for *AabHLH1*. PCR-amplified products were cloned into the T/A cloning vector pMD18-T (TaKaRa, Dalian, China) and sequenced. The full length of the amino acid sequences deduced for AaMYB3 and AabHLH1 were constructed using the ClustalX2 and the MEGA 5.05 programs (the Neighbor-Joining method with 1000 bootstrap replications) for sequence alignment and phylogenetic analysis, respectively.

### Quantitative real-time PCR (q-PCR) analysis

The q–PCR analysis was conducted as previously described^29^. The relative expression of the anthurium genes was normalized to the expression of the cyclophilin gene (*AaCYP*) and ubiquitin family protein gene (*AaUBQ5*) as described by Gopaulchan et al.^36^. The *AaANR* (GenBank accession no. MH349478) and *AaLAR* (MH349479) nucleotide sequences were obtained from our transcriptome database^29^, which shared extremely high identity in the open reading frame (ORF) (99.4% and 99.2%) with the related species *Anthurium amnicola ANR* (GDJX01012972.1) and *LAR* (GDJX01014607.1), respectively.

The relative expression of the tobacco genes was normalized to the expression of the actin gene (*NbACT*) and the ribosomal protein L25 gene (*NtL25*) as described by Pérez-Díaz et al.^37^. The relative expression of the *Arabidopsis* genes was normalized to the expression of *AtActin* and *AtUBQ1* as described by Matsui et al.^38^. and Han et al.^39^. The sequences of all the primers used in q-PCR were listed in Table S1. Triplicates of each reaction were performed.

### Overexpression vector construction and plant transformation

The fragments of *AaMYB3* and *AabHLH1* that contain the ORF were separately cloned into the plant binary vector pCXSN (T-Vector) for constitutive gene expression as described by Chen et al.^40^. The resulting vector pCXSN-*AaMYB3* and pCXSN-*AabHLH1* were transferred into *Agrobacterium tumefaciens* strain EHA105 using the freeze–thaw method, which was used in plant transformation. The genetic transformation of the *Arabidopsis* plants was based on the floral dip method^41^. The *Agrobacterium* strain EHA105 harboring the recombinant plasmids pCXSN-*AaMYB3* was used to transform the *Arabidopsis tt2* mutant plants, and *AabHLH1* was transformed into the *Arabidopsis tt8* mutant plants. Seeds harvested from the transformed plants (T_0_) were germinated on 1/2 MS media containing 35 mg/L hygromycin. Positive homozygous progenies were selected and confirmed using genomic PCR. The seeds of the T_3_ progeny *Arabidopsis* plants were observed.

The genetic transformation of tobacco with *AaMYB3* and *AabHLH1* was accomplished using *Agrobacterium*-mediated transformation^42^. The transgenic plants were selected by hygromycin resistance and once rooted were transferred to soil and grown in the greenhouse. Positive transgenic T_1_ progeny tobacco plants identified by genomic PCR and q-PCR were used for further studies. Transgenic tobacco plants (T_0_ progeny, three lines of each gene transformation) separately expressing *AaMYB3* and *AabHLH1* were crossed in both the ♀ × ♂ and ♂ × ♀ directions. The F_1_ seeds of the AaMYB3 × AabHLH1 plants were harvested and germinated. The seedlings were selected using genomic PCR and q-PCR, and a total of 49 plants expressing both AaMYB3 and AabHLH1 were obtained for further studies.

### Yeast two-hybrid (Y2H) assay

The Y2H assay to investigate the interactions between AaMYBs and AabHLH1 was performed as described by Nakatsuka et al.^43^. The full-length CDS of *AaMYB3* and *AaMYB2* or *AabHLH1* were cloned into the pGADT7 vector or pGBKT7 vector (Clontech, Mountain View, US), respectively. The yeast transformation and clones selection were performed as previously described^44^. The interaction of AtMYB75 and AtTT8 from *Arabidopsis* was employed as a positive control^45^.

### Bimolecular fluorescence complementation (BiFC) assay

The full-length CDS of *AaMYB3* and *AaMYB2* or *AabHLH1* were cloned into the binary yellow fluorescent protein (YFP) BiFC vectors pXY106 (nYFP) or pXY104 (cYFP)^46^, respectively, resulting in the recombinant plasmids AaMYB3-nYFP, AaMYB2-nYFP, and AabHLH1-cYFP. The empty vectors and the recombinant plasmids were transferred into *A*. *tumefaciens* GV3101 using the freeze–thaw method and were transiently expressed in *Nicotiana benthamiana* leaf by agroinfiltration. Two days after transformation, the YFP signals were examined in the transfected cells using a Zeiss780 confocal microscope (Zeiss, Jena, Germany). The gene AT1G16610 (encoding SR45) from *Arabidopsis* was coexpressed as a fusion to the red fluorescent protein (RFP) with the target genes in *N*. *benthamiana* leaf cells and was used as a nuclear localization marker^47^. The interaction of AtMYB75 and AtTT8 from Arabidopsis was employed as a positive control^45^.

### DMACA Staining

Dry *Arabidopsis* seeds were stained with the dimethylaminocinnamaldehyde (DMACA, Sigma) reagent (2% [w/v] DMACA in 3 M HCl/50% [w/v] methanol) for 2 days and washed several times with 70% ethanol (v/v) as described by Abrahams^48^. The stained seeds were photographed using a Leica M205FA microscope equipped with a DFc450c camera (Leica Microsystems, Solms, Germany).

## Results

### Sequences of *AaMYB3* and *AabHLH1* and a phylogenetic analysis

Several unigenes associated with the transcriptional control of the flavonoids and PA biosynthesis were selected from our transcriptome data according to annotations determined with BLASTx searches in the National Center for Biotechnology Information (NCBI) Nr and Swiss-Prot protein databases (data not shown). Among these, one unigene with a length of 1042 bp containing an 891-bp ORF matched AtTT2, which is specific to the PA pathways in *Arabidopsis*, and one unigene with a length of 2694 bp containing a 2115-bp ORF matched a bHLH activator of PA synthesis in *Arabidopsis*, AtTT8. Therefore, these two candidate proteins may play roles in the modulation of PA biosynthesis in anthurium. The ORFs of the MYB and bHLH TF genes were amplified using RT-PCR from the spathe of the cultivar “Tropical” sampled at developmental stage 2. The two genes were designated *AaMYB3* and *AabHLH1*, respectively, and encoded proteins of 297 and 705 amino acids, respectively.

**Fig. 1:**
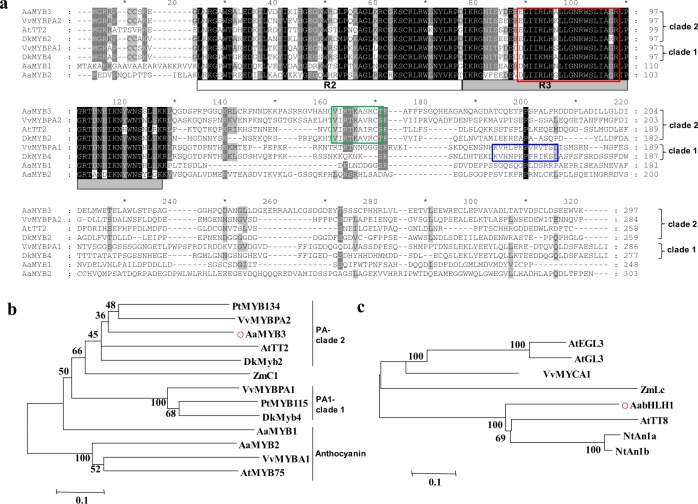
Sequence alignments of AaMYB3, phylogenic tree of AaMYB3 and AabHLH1 with known R2R3-MYBs and bHLHs involved in proanthocyanidin (PA) biosynthesis. **a** Alignment of the full-length deduced amino acid sequence of AaMYB3 with the other R2R3-MYBs. The R2 and R3 domains are shown; the bHLH interacting motif are indicated in the red box; the conserved motifs VI[R/P]TKAx1RC[S/T] for the PA-regulating MYBs PA-clade 2 are indicated in the green box, and the conserved motifs K[I/V]x2PKPx1Rx2S[I/L] for PA-clade 1 are indicated in the blue box. Identical nucleotides are shown on a black background, and gaps are indicated by dashes. **b** Phylogenetic relationship of AaMYB3 with selected known PA and anthocyanin MYB regulators from anthurium and other species. The scale bar represents 0.1 substitutions per site. **c** Phylogenetic relationship of AabHLH1 with selected known bHLH regulators involved in PA and anthocyanin biosynthesis and trichome development from other species. The scale bar represents 0.1 substitutions per site. The MYB names and GenBank accession numbers are as follows: AaMYB3, MH349476, AaMYB1, AAO92352.1, AaMYB2, AML84515 (*Anthurium andraeanum*); PtMYB134, ACR83705.1, PtMYB115, XM 002302608.2 (*Populus* spp.);VvMYBPA1, CAJ90831.1, VvMYBPA2, ACK56131.1, VvMYBA1, BAD18977.1 (*Vitis vinifera*): AtTT2, CAC40021.1, AtMYB75, NP 176057.1 (*Arabidopsis thaliana*); DkMyb2, AB503699, DkMyb4, AB503701 (*Diospyros kaki*); ZmC1, 1613412E (*Zea mays*); bHLH names and GenBank accession numbers are as follows: AabHLH1, MH349477; AtEGL3, AEE3412, AtGLABRA3, AED94664.1, AtTT8, AEE82802.1 (*Arabidopsis thaliana*); VvMYCA1, NP 001267954.1 (*Vitis vinifera*); ZmLc, NP 001105339.1 (*Zea mays*); NtAn1a, AEE99257.1, NtAn1b, and AEE99258.1 (*Nicotiana tabacum*)

An analysis of the deduced amino acid sequence of AaMYB3 suggested that an N-terminal R2R3 repeat corresponds to the DNA-binding (MYB) domain. Similarly to other related MYBs, the bHLH interaction motif [D/E]Lx2[R/K]x3Lx6Lx3R was identified in the highly conserved N-terminal R2R3 region of AaMYB3. In an alignment analysis with MYBs known to function in the regulation of PA biosynthesis and an additional two anthurium anthocyanin MYB regulators, we identified the C-terminal motif conserved in the AtTT2-like MYBs, VI[R/P]TKAx_1_RC[S/T], in AaMYB3. That indicates that AaMYB3 is very closely related to VvMYBPA2, AtTT2, and DkMYB2 and belongs to the PA-clade 2 MYB regulators (Fig. 1a). The conversed motif K[I/V]x2PKPx1Rx2S[I/L] is only found in the PA-clade 1 MYBs, such as VvMYBPA1 and DkMYB4. The two PA-regulating motifs are not identified in the other two anthurium anthocyanin regulatory MYBs identified, AaMYB1 and AaMYB2. In a phylogenetic analysis, AaMYB3 clustered with AtTT2, VvMYBPA2, and PtMYB134 in PA-clade 2, and AaMYB1 and AaMYB2 from anthurium clustered with the anthocyanin regulatory MYBs AtMYB75 and VvMYBA1 (Fig. 1b). These sequence and phylogenetic analyses suggest that AaMYB3 plays a role in PA biosynthesis.

A sequence analysis of AabHLH1 showed the following conserved motifs: the MYB interaction region at the N-terminal; the bHLH domain in the C-terminal region, and the transactivation (ACT) domain (Fig. S1). In a phylogenetic analysis, AabHLH1 clustered with AtTT8 and NtAn1a/NtAn1b, which are involved in the biosynthesis of flavonoids, such as PAs and anthocyanins (Fig. 1c). These results suggest that AabHLH1 participates in the biosynthesis of the PAs or other flavonoids in anthurium.

### *AaMYB3* is predominately expressed in the spathe and negatively correlates with anthocyanin accumulation in the spathes of cultivars with different color phenotypes

**Fig. 2:**
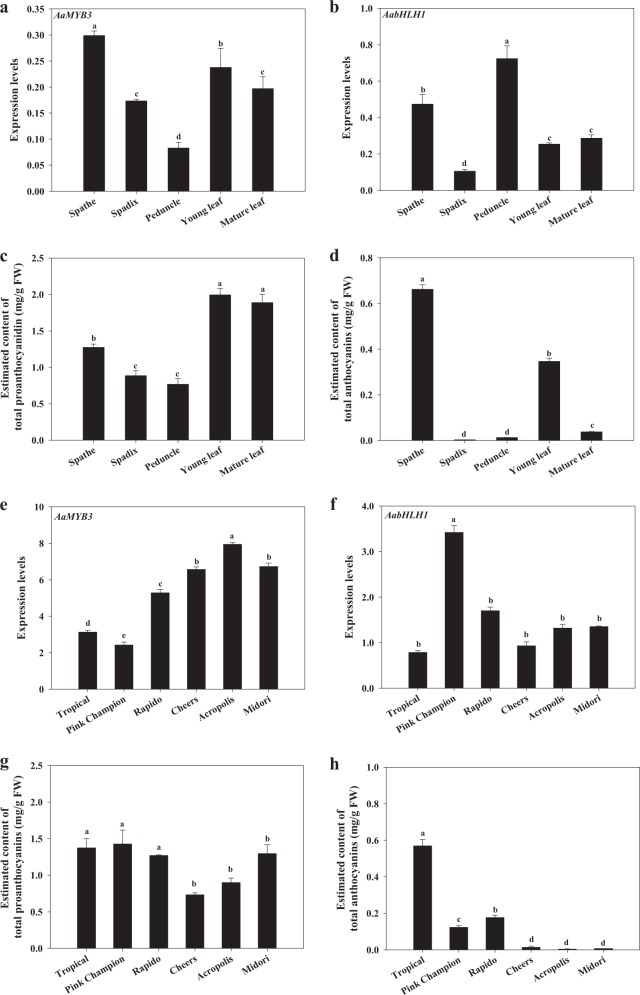
The expression pattern of *AaMYB3* and *AabHLH1*, as well as the content of total proanthocyanidins and anthocyanins, in anthurium. The expression of *AaMYB3* (**a**) and *AabHLH1* (**b**), the content of total proanthocyanidins (**c**) and anthocyanins (**d**) in various tissues of the cultivar “Tropical”; The expression level of *AaMYB3* (**e**) and *AabHLH1* (**f**), the content of total proanthocyanidins (**g**) and anthocyanins (**h**) in the spathes of different cultivars with various color phenotypes. The data were presented as the mean ± SD (*n* = 3). Values with different letters are significantly different according to Duncan’s multiple range tests at the 5% level

To explore the transcript profiles of the new MYB and bHLH genes, cDNA samples from different tissues of anthurium were analyzed using q-PCR. We detected the transcripts of *AaMYB3* and *AabHLH1* in various anthurium tissues with no obvious tissue specificity. The *AaMYB3* transcripts were predominantly detected in the spathe, followed by the leaves and then the spadix (Fig. 2a). The order of the *AabHLH1* expression from the highest to lowest was as follows: peduncle > spathe > mature leaf and young leaf > spadix (Fig. 2b). Factoring in the PA and anthocyanin contents of various tissues, the relative expression levels of *AaMYB3* were high in the spathes and leaves where PA primarily accumulates (Fig. 2c). The *AaMYB3* expression is associated with anthocyanin accumulation in various “Tropical” tissues (Pearson Correlation Coefficient, *r* = 0.832^**^, *P* < 0.01) (Fig. 2d). However, the expression pattern of *AabHLH1* was not related to the accumulation pattern of the PA or the anthocyanin (Fig. 2c, d).

To further investigate the correlation between the expression of *AaMYB3* and *AabHLH1* and the amounts of flavonoid present, the expression levels of the two TF genes and the PA and anthocyanin contents were determined in spathes with red, pink, purple, pale-pink, white, and green phenotypes. *AaMYB3* and *AabHLH1* were expressed in the spathes of all the cultivars tested where PAs also accumulated (Fig. 2e–g). Notably, anthocyanins accumulated primarily in the red spathe (Tropical) and then in the purple (Rapido) and pink spathes (Pink Champion) (Fig. 2h). These results further suggest that the expression of *AaMYB3* is negatively related to anthocyanin accumulation in the spathe of anthurium cultivars with various color phenotypes (*r* = ‒0.669^**^, *p* *<* 0.01).

### Complementation of the *Arabidopsis tt8* and *tt2* mutants by the ectopic expression of *AabHLH1* and *AaMYB3*

**Fig. 3:**
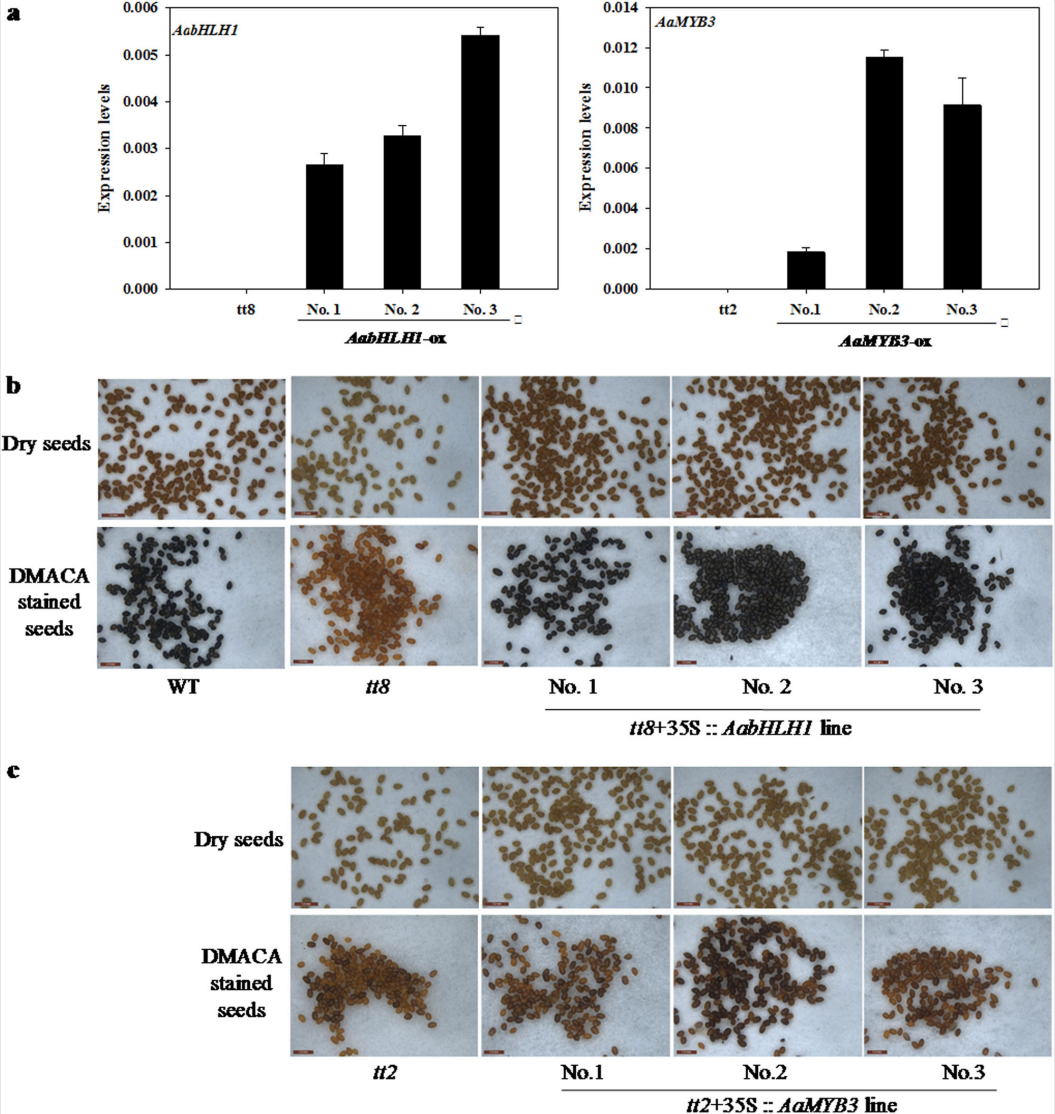
Complementation analysis of *Arabidopsis tt8* and *tt2* mutants by the overexpression of *AabHLH1* and *AaMYB3*. **a** q–PCR test for the expression of *AabHLH1* in *AabHLH1*-overexpression (ox) lines No. 1, No. 2, and No. 3, and the expression of *AaMYB3* in *AaMYB3*-ox lines No.1, No. 2, and No. 3 using the leaf samples of the *tt8* and *tt2* mutant and transgenic lines. **b** Dry (upper panel) and DMACA-stained (bottom panel) seeds of the wild-type, *tt8* mutant, and *AabHLH1*-ox lines No. 1, No. 2, and No. 3. **c** Dry (upper panel) and DMACA-stained (bottom panel) seeds of the wild-type, *tt2* mutant, and *AaMYB3*-ox lines No. 1, No. 2, and No. 3

To test whether AabHLH1 and AaMYB3 function as PA regulators, the two genes were introduced into the *tt8* and *tt2* mutants, respectively, and expressed under the control of the cauliflower mosaic virus 35S (CaMV35S) promoters (Fig. 3a). The transgenic T_3_ seeds of the *AabHLH1*-*tt8* line had a brown seed coat, which was stained black by the DMACA reagent such as the wild type (Fig. 3b). This result demonstrates that the overexpression of *AabHLH1* complemented the *tt8* mutant seed coat phenotype. The color of the T_3_ seed coat in the *AaMYB3*-*tt2* line was slightly darker than that of the *tt2* mutant before and after DMACA staining (Fig. 3c). Although the restoration of the seed coat phenotype by *AaMYB3* complementation was not as obvious as during the *AabHLH1* complementation in the *tt8* mutant, the expression of the PA-specific enzyme gene *AtANR* was clearly upregulated in all the transgenic *AaMYB3*-*tt2* lines (Fig. S2).

### Effects of *AaMYB3* and *AabHLH1* overexpression in tobacco

**Fig. 4:**
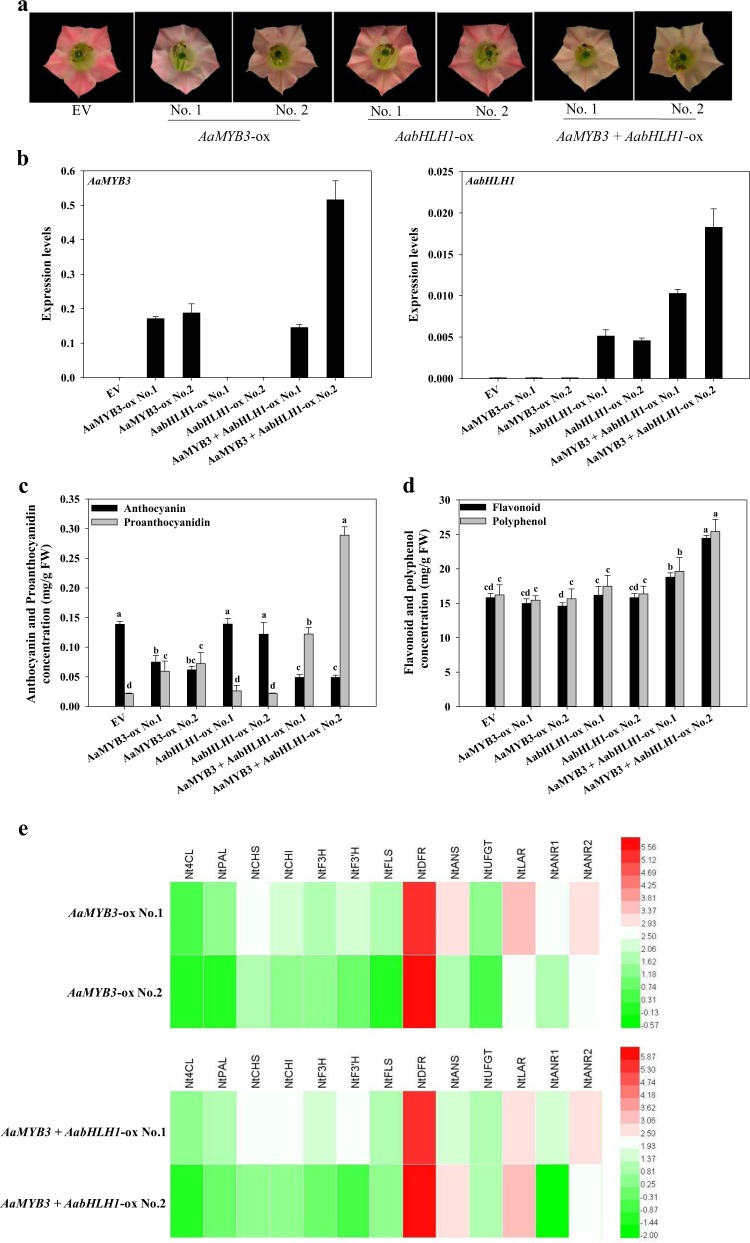
Typical phenotypes, secondary metabolite amounts, and gene expression of the transgenic tobacco corolla limbs. **a** Typical floral phenotypes of the EV (empty vector transformed line), *AaMYB3*-overexpression (ox) lines No. 1 and No. 2, *AabHLH1*-ox lines No. 1 and No. 2, and *AaMYB3* + *AabHLH1*-ox lines No. 1 and No. 2. **b** *AaMYB3* and *AabHLH1* expression in the EV and transgenic tobacco corolla limbs tested using q-PCR. **c** Total anthocyanin and proanthocyanidin contents in the corolla limbs of the EV and transgenic tobacco lines. **d** Total flavonoid and polyphenol contents in the corolla limbs of the EV and transgenic tobacco lines. **e** The relative expression analysis of the flavonoid biosynthetic genes in the tobacco corolla limbs of *AaMYB3*-ox lines and *AaMYB3* + *AabHLH1*-ox lines. Color bar: Log_2_ (fold changes). The data are presented as the mean ± SD (*n* = 3). Values with different letters are significantly different according to Duncan’s multiple range tests at the 5% level

To investigate whether the ectopic expression of *AaMYB3* and *AabHLH1* affects PA biosynthesis, we analyzed transgenic tobacco plants that independently overexpressed *AaMYB3* or *AabHLH1* and simultaneously overexpressed both *AaMYB3* and *AabHLH1* (*AaMYB3* + *AabHLH1*). Two independent T_1_ transgenic lines from each construct were selected based on their levels of transgene expression (Fig. 4b). Under the same growing conditions, the corolla limb colors of the T_1_ *AaMYB3*-overexpressing (ox) lines changed to light pink compared with the control plants transformed with the empty vector (EV). The corolla limb color of the T_1_ *AabHLH1*-ox lines showed no obvious phenotypic changes relative to the EV control. Interestingly, the corolla limbs of the T_1_ *AaMYB3* + *AabHLH1*-ox plants were much paler pink than either the *AaMYB3*-ox plants or the EV control plants (Fig. 4a). An analysis of the anthocyanin contents showed changes that paralleled those in the color phenotype. The levels of total anthocyanins from the lowest to highest were as follows: *AaMYB3* + *AabHLH1*-ox lines < *AaMYB3*-ox lines < EV or *AabHLH1*-ox lines. The PA contents in the tobacco corolla limbs correlated negatively with the anthocyanin content. The *AaMYB3* + *AabHLH1*-ox lines showed the highest PA content, and the *AaMYB3*-ox lines also showed markedly higher PA content in the corolla limbs than the EV or *AabHLH1*-ox lines (Fig. 4c). The total flavonoid and polyphenol contents were also measured. Only the *AaMYB3* + *AabHLH1*-ox lines showed higher total flavonoid and polyphenol contents than the other lines (Fig. 4d). These results suggest that the overexpression of *AaMYB3* significantly enhanced the accumulation of the PAs leading to a pale pigmentation in the corolla limb of the transgenic plants. The PA regulatory function of AaMYB3 was increased synergistically by AabHLH1. Simple *AabHLH1* overexpression had no significant effect on the anthocyanin or PA in the transgenic tobacco corolla limbs.

A q-PCR analysis was performed to further analyze the effects of the ectopic expression of *AaMYB3* and *AaMYB3* + *AabHLH1* on the target enzyme genes involved in the biosynthesis of flavonoids in tobacco. The overexpression of *AaMYB3* alone affected the expression of the anthocyanin- and PA-related genes in the transgenic lines, and in particular, clearly upregulated the expression of the late anthocyanin enzyme genes *NtDFR* and *NtANS* and the key PA biosynthetic genes *NtLAR* and *NtANR* (Fig. 4e). In the *AaMYB3* + *AabHLH1*-ox lines, the expression pattern of the genes in the flavonoid biosynthesis pathway was similar to that in the *AaMYB3*-ox lines (Fig. 4e). These data indicate that AaMYB3 alone or together with AabHLH1 upregulates or activates the expression of key anthocyanin and PA biosynthetic key genes, ultimately promoting PA accumulation in transgenic tobacco.

### Interaction of AabHLH1 with different AaMYBs partners

**Fig. 5:**
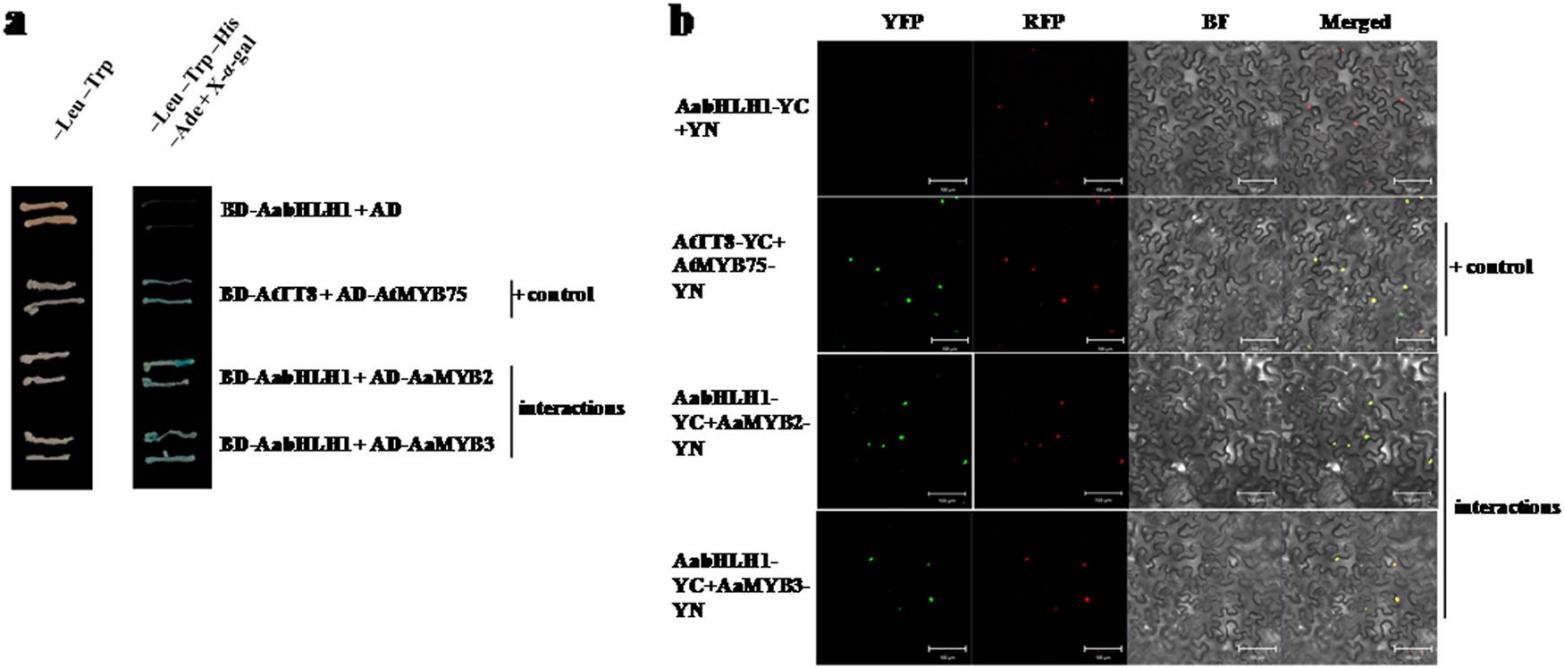
Interactions between AabHLH1 and AaMYB2 and AaMYB3 detected using the Y2H and BiFC assays. **a** Y2HGold yeast cells containing plasmids pGADT7 + pGBK-AabHLH1, pGAD-AaMYB2 + pGBK-AabHLH1 or pGAD-AaMYB3 + pGBK-AabHLH1were grown on double- and quadruple-selection media, and pGAD-AtMYB75 + pGBK-AtTT8 was used as the positive control. The X-*α*-gal assay was performed to confirm the positive interactions. **b** Bimolecular fluorescence complementation visualization of the AabHLH1 and AaMYB2 and AaMYB3 interaction in the *N. benthamiana* leaf epidermal cells. The AtMYB75-AtTT8 interaction was used as a positive control. YFP yellow fluorescent protein field, RFP red fluorescent protein, which indicated the nuclear localization of *Arabidopsis* SR45; BF bright field. Merged, overlay of the YFP, RFP, and BF field. Bars, 100 μm

The MBW ternary transcription complex is usually formed to regulate flavonoid biosynthesis in plants. The interaction of AabHLH1 with AaMYB3 and the previously reported anthocyanin regulator AaMYB2 was investigated using a Y2H assay. The autoactivation of pGBK-AaMYB3 or pGBK-AaMYB2 was observed on SD/–Trp/–His/–Ade media containing 5-bromo-4-chloro-3-indolyl-*α*-d-galactopyranoside (X-*α*-Gal). Therefore, *AaMYB3* or *AaMYB2* was fused to the *GAL4* DNA-activating domain, and *AabHLH1* was fused to the *GAL4* DNA-binding domain. As shown in Fig. 5a, the protein–protein interactions between AabHLH1 and AaMYB3 or AaMYB2 were demonstrated by the growth of colonies containing both the pGAD-AaMYB3 and pGBK-AabHLH1 vectors or both the pGAD-AaMYB2 and pGBK-AabHLH1 vectors on SD/–Leu/–Trp/–His/–Ade + X-*α*-Gal media. Thus, the Y2H assay suggested that AabHLH1 interacts with both AaMYBs and forms a transcriptional complex.

The interaction of AabHLH1 with AaMYBs was confirmed using a BiFC assay. AabHLH1 tagged with the split cYFP fragment (YC) and AaMYB3 or AaMYB2 tagged with the split nYFP fragment (YN) were transiently coexpressed in *N. benthamiana* leaves. As shown in Fig. 5b, the YFP fluorescent signal was detected in tobacco epidermal cells expressing both the AaMYB3-YN and AabHLH1-YC fusion proteins and the AaMYB2-YN and AabHLH1-YC fusion proteins, and they successfully merged with the RFP fluorescent signals, while no YFP fluorescent signal was detected in the epidermal cells expressing AabHLH1-YC with only YN. The BiFC assay demonstrated the interaction between AabHLH1 and AaMYB2 and AaMYB3 *in vivo*, and their protein-protein complexes were localized in the nucleus.

### Expression trends in *AaMYB3* and *AabHLH1* are coincident with those of several anthocyanin and PA biosynthetic genes

**Fig. 6:**
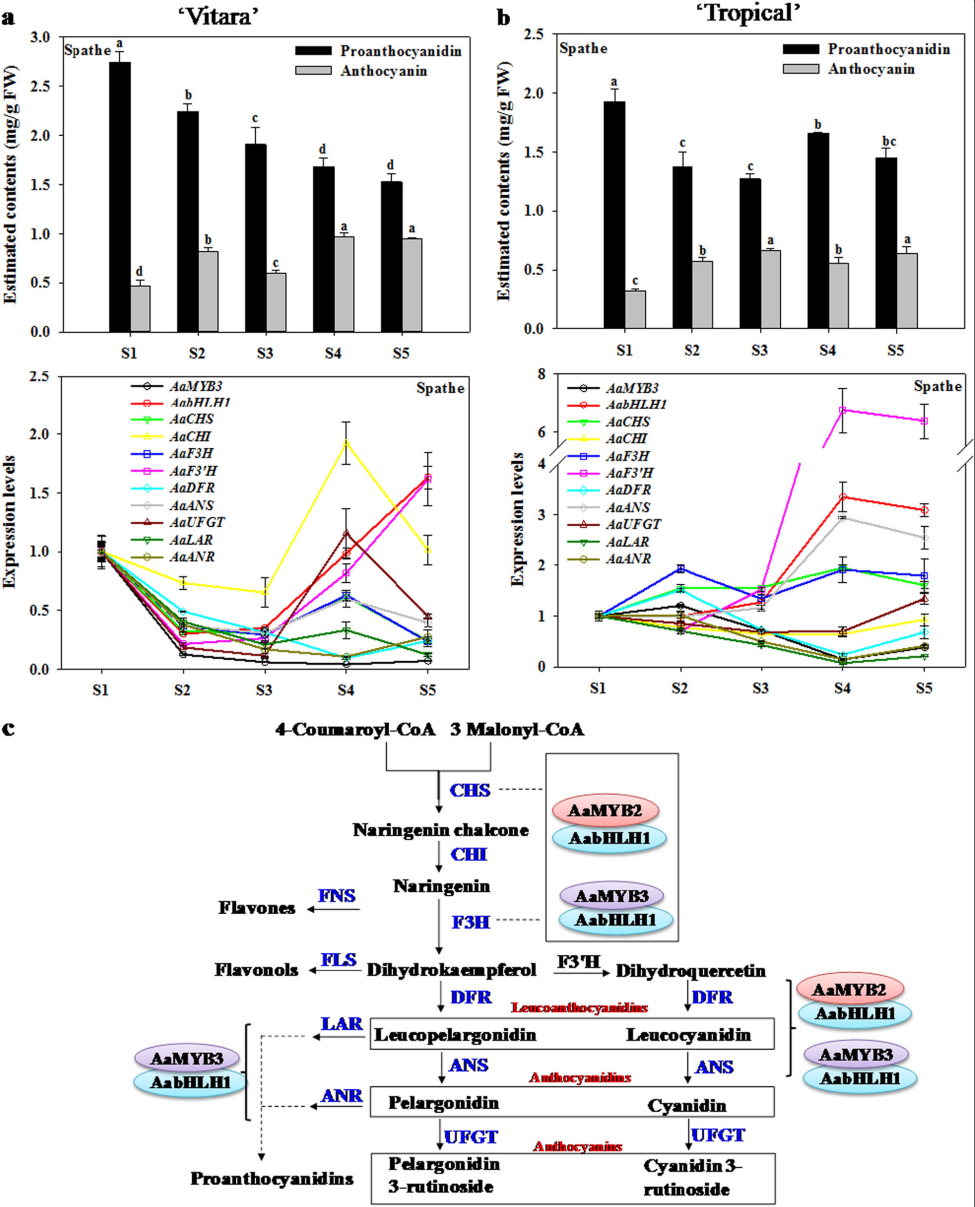
Analysis of the function of AaMYB3 and AabHLH1 in anthurium. The detection for the content of total proanthocyanidins and anthocyanins, as well as the expression of *AaMYB3*, *AabHLH1*, and the flavonoid biosynthetic genes in the developmental spathe of cultivars “Vitara” (**a**) and “Tropical” (**b**). The data were presented as the mean ± SD (*n* = 3). Values with different letters are significantly different according to Duncan’s multiple range tests at the 5% level. **c** A proposed model for the regulation of proanthocyanin and anthocyanin biosynthesis by AaMYB2, AaMYB3, and AabHLH1 in the anthurium spathe

The ectopic expression *AaMYB3* alone or the coexpression of *AaMYB3* and *AabHLH1* in tobacco stimulated PA production by activating a number of anthocyanin and PA biosynthetic genes. Therefore, AaMYB3 and AabHLH1 are hypothesized to act as PA activators in anthurium. To investigate the possible regulatory roles of AaMYB3 and AabHLH1, a gene coexpression analysis was performed in the developing spathes and spadices of the cultivars “Vitara” and “Tropical”, because they are the primary ornamental organs, and *AaMYB3* is predominantly expressed in the spathe. The spathes of both cultivars simultaneously contained anthocyanins and PAs in all the developmental stages investigated (Fig. 6a, b). As shown in Fig. 6a, the expression of *AaMYB3* decreased progressively during development in the spathe of the cultivar “Vitara”, and the expression of *AabHLH1* decreased in stage 2 and then increased again and peaked in stage 5. The expression pattern of *AaMYB3* appeared to be related to PA accumulation (*r* *=* 0.840^**^, *p* < 0.01) and negatively related to anthocyanin accumulation (*r* = –0.741^**^, *p* *<* 0.01) (Fig. 6a). Of the anthocyanin and PA biosynthetic genes, the early flavonoid synthetic genes *AaCHS* and *AaF3H*, the anthocyanin-specific genes *AaDFR* and *AaANS* and the PA-specific genes *AaLAR* and *AaANR* were coordinately expressed with *AaMYB3* (Fig. 6a). The correlation coefficients are shown in Table S2. However, unlike *AaMYB3*, the expression of *AabHLH1* showed no obvious relationship with either PA or anthocyanin accumulation and was only strongly coexpressed with *AaF3′H* (*r* = 0.952^**^, *p* < 0.01). In the spathe of the cultivar “Tropical”, the expression of *AaMYB3* peaked at stage 2 before subsequently decreasing, reaching its lowest level in stage 4 and then increased again in stage 5 (Fig. 6b). The expression of *AabHLH1* increased gradually and peaked in stage 4 after which it decreased slightly (Fig. 6b). The expression patterns of *AaMYB3* and *AabHLH1* in the ‘Tropical’ spathe were not associated with anthocyanin or PA accumulation (Fig. 6a, b). The correlation analysis suggests that *AaMYB3* coexpressed with *AaDFR*, *AaLAR*, and *AaANR* and that *AabHLH1* is coexpressed with *AaCHS*, *AaF3H*, *AaF3′H*, and *AaANS* in the “Tropical” spathe (Fig. 6b, Table S2).

The spadix of cultivar “Vitara” accumulated both anthocyanins and PAs at all the development stages investigated (Fig. S3a), while the “Tropical” spadix accumulated only PAs during its development (Fig. S3b). The expression pattern of *AaMYB3* showed no obvious correlation with either anthocyanin or PA accumulation in the spadix of “Vitara” (Fig. S3a). Similar to that in the spathe, *AaMYB3* was coexpressed with most of the genes in the anthocyanin and PA biosynthetic pathways, such as *AaCHS*, *AaCHI*, *AaF3H*, *AaDFR*, *AaANS*, and *AaLAR* (Fig. S3a, Table S2). *AabHLH1* expression positively correlated with that of *AaUFGT* and *AaANR* (Fig. S3a, Table S2). In the “Tropical” spadix, *AaCHS*, *AaF3H*, *AaDFR*, *AaANS*, *AaLAR*, and *AaANR* showed a similar expression pattern to that of *AaMYB3*, and *AaCHI*, *AaF3′H*, and *AaUFGT* were coexpressed with *AabHLH1* (Fig. S3b). These results suggest that AaMYB3 may target several genes in the anthocyanin and PA biosynthetic pathways and activate PA biosynthesis in anthurium. AabHLH1 may play a role in the whole flavonoid biosynthetic pathway and is not specific to PA biosynthesis.

## Discussion

PAs are colorless flavonoid polymers that accumulate in plants and are usually controlled by the MBW TF complex^5^. In this study, we report the isolation and analysis of the R2R3-MYB gene *AaMYB3* and bHLH TF gene *AabHLH1* from anthurium. The AaMYB3 protein is similar to the known PA regulators grape VvMYBPA2, *Arabidopsis* AtTT2, poplar PtMYB134, and persimmon DkMyb2, which share a short conserved VI[R/P]TKAx_1_RC[S/T] motif^10^. According to Zhu et al.^21^, MYB PA regulators can be categorized into two subgroups: PA-clade 1 includes VvMYBPA1 and DkMyb4, and PA-clade 2 includes AtTT2, VvMYB2, PtMYB134, and DkMyb2. The two types of PA regulators display different expression patterns and recognize different types of motifs in the promoter regions of the PA pathway genes^21^. According to our phylogenetic analysis, AaMYB3 clustered in the PA-clade 2 subgroup^21^, clearly distinct from the previously reported anthocyanin regulators AaMYB1^27^ and AaMYB2^29^. AabHLH1 is homologous with *Arabidopsis* AtTT8 and tobacco NtAn1, which are members of the bHLH TF subgroup IIIf, which is involved in the regulation of PA and flavonoid biosynthesis^8,49,50^. Our amino acid sequence analysis suggested that AaMYB3 contains the bHLH interaction motif and that AabHLH1 contains the MYB interaction region. Therefore, it is possible that the two proteins physically interact. These results imply that these two TF are involved in the PA biosynthetic pathway.

In a previous study, we showed that the expression pattern of *AaMYB2* correlates strongly with anthocyanin accumulation^29^. The expression pattern of *AaMYB3* in the different tissues of anthurium cultivar “Tropical” was similar to that of the anthocyanin regulator gene *AaMYB2*, which was predominantly expressed in the spathe and exhibited a positive correlation with anthocyanin accumulation. However, unlike *AaMYB2*, the expression of *AaMYB3* exhibited a negative correlation with anthocyanin accumulation in the spathes of different anthurium cultivars with various color phenotypes. It should be noted that *AaMYB3* is more strongly expressed in the green and white spathes where proanthocyanin accumulates, and no anthocyanin was detected. This indicated that the expression pattern of *AaMYB3* in anthurium may be associated with PA accumulation. This was confirmed in the developing “Vitara” spathes in which the expression pattern of *AaMYB3* was exactly consistent with the accumulation of PA and correlated negatively with anthocyanin accumulation. Similar results have been described for PA regulators in grapevine (VvMYBPA1^13^) and coleus (SsMYB3^21^).

Because AaMYB3 is homologous to AtTT2, a complementation test of the *Arabidopsis tt2* mutant was used to functionally characterize AaMYB3. In the *Arabidopsis tt2* and *tt8* mutants, the AtTT2–AtTT8–AtTTG1 ternary transcription complex fails to form, and the expression of the PA biosynthesis gene *AtANR* is not induced^8^. The lack of PAs in the two mutants causes a yellow seed phenotype, and the overexpression of a homologous gene restored the seed color. Although AaMYB3 clustered in PA-clade 2, the overexpression of *AaMYB3* caused only a slight recovery of the *tt2* mutant seed coat phenotype. This was not as marked as the effects of other PA-clade 2 MYBs, such as AtTT2^10^ and VvMYBPA1^13^. To analyze the function of AaMYB3 in the *tt2* mutant, the expression of the genes in the PA pathway was detected in the leaves of the T_3_ transgenic *AaMYB3*-*tt2* plants. The expression of the PA-specific *AtANR* was significantly upregulated compared with that in the *tt2* mutant control. This implied that AaMYB3 activates the expression of *AtANR* to some degree. However, this degree of activation is insufficient to cause enough PA accumulation in the seed coat and therefore, to completely complement the mutant phenotype. In the transcriptional regulation of PA biosynthesis in *Arabidopsis*, TT2 is responsible for the specific recognition of the promoter of *AtANR* in combination with TT8^8^. In the *AaMYB3*-*tt2* transgenic line, AaMYB3 might not have combined well with AtTT8 to efficiently activate PA biosynthesis. The function of AaMYB3 was also characterized by its ectopic expression in tobacco. The overexpression of *AaMYB3* alone produced light pink corolla limbs with greatly accumulated PAs. This is consistent with the overexpression of other PA-related MYB regulators in tobacco, coleus SsMYB3^21^, grapevine VvMYBPA1^51^, and *Malus crabapple* McMYB12b^22^. The functions of these MYBs are thought to be conserved across a diverse range of species. The overexpression of *AaMYB3* in the corolla limbs of transgenic tobacco caused the significantly upregulated expression of the key genes in the anthocyanin (*NtDFR* and *NtANS*) and PA biosynthetic pathways (*NtLAR* and *NtANR*). This result is similar to the results when *SsMYB3*^21^ and *VvMYBPA1*^51^ were overexpressed in the transgenic tobacco flowers and shows that AaMYB3 is functionally homologous to the MYBs described above, which are involved in the regulation of PA biosynthesis.

The expression pattern of *AabHLH1* was not associated with either PA or anthocyanin in various anthurium tissues, the spathes of different cultivars, or at different developmental stages of the spathe. This is consistent with the expression of the anthocyanin-modulating *PabHLH3* in sweet cherry^52^ but differs from the bHLH-TF-controlled anthocyanin biosynthesis in other horticultural plants, such as *LcbHLHs* in litchi^53^ and *DhbHLH1* in dendrobium^44^. It can be hypothesized that the expression of *AabHLH1* in anthurium is not directly involved in the accumulation of the PA or anthocyanin pigments. Our phylogenetic analysis indicates that AabHLH1 is homologous to AaTT8. Therefore, a complementation test of the *Arabidopsis tt8* mutant was performed to functionally characterize AabHLH1. As expected, the overexpression of *AabHLH1* in the *tt8* mutant complemented the seed coat phenotype. In the *AabHLH1*-*tt8* transgenic line, AabHLH1 functioned as AtTT8, combined with AtTT2 and successfully activating the PA pathway. This suggests that AabHLH1 plays a role in PA biosynthesis. When *AabHLH1* was overexpressed in tobacco, it caused no significant change in the phenotype or in the accumulation of the anthocyanins or PAs in the corolla limbs. This outcome is similar to the phenomenon observed when tobacco was transformed with the *MrbHLH1* of Chinese bayberry. The transgenic tobacco line overexpressing *MrbHLH1* showed a phenotype similar to that of the control^54^. However, the line overexpressing both *MrbHLH1* and *MrMYB1* accumulated significant amounts of anthocyanin in the whole plant^54^. Therefore, we hypothesize that AabHLH1 requires an appropriate MYB partner to participate in PA biosynthesis. A transgenic tobacco line overexpressing both *AabHLH1* and *AaMYB3* was obtained by crossing transgenic plants that separately overexpressed *AaMYB3* and *AabHLH1* as described by Xie et al.^55^. The offspring of the cross, which coexpressed *AaMYB3* and *AabHLH1*, had much paler pink corolla limbs than the *AaMYB3*-ox line, and they accumulated PAs strongly, as well as total flavonoids and polyphenols. As in the *AaMYB3*-ox line, the tobacco anthocyanin and PA biosynthetic genes *NtDFR*, *NtANS*, *NtLAR*, and *NtANR* were strongly upregulated in the *AaMYB3* + *AabHLH1*-ox line. As a result, in the transgenic tobacco, AabHLH1 recognized AaMYB3 as its partner and enhanced the expression of *AaMYB3* for efficient PA biosynthesis. The formation of the AaMYB3–AabHLH1 and AaMYB2–AabHLH1 protein complex was confirmed using the Y2H and BiFC assays. These provided further evidence of the involvement of AabHLH1 in PA biosynthesis via its physical interaction with AaMYB3. There was evidence that AabHLH1 interacts with AaMYB2, which was identified as an anthocyanin regulator in anthurium in our previous study^29^. Therefore, AabHLH1 may also play a role in anthocyanin biosynthesis.

The PA regulatory functions of *AaMYB3* and *AabHLH1* were characterized by their exogenous expression in *Arabidopsis* and tobacco. However, the regulatory mechanism controlling PA production in anthurium is still unclear. Because *AaMYB3* is primarily expressed in the spathe, our gene expression analysis was primarily performed in spathe tissues. In the red spathes of cultivars “Vitara” and “Tropical”, the expression pattern of *AaMYB3* was similar to that of *AaCHS* (*r* = 0.845^**^, *p* < 0.01), *AaDFR* (*r* = 0.924^**^, *p* < 0.01), *AaF3H* (*r* = 0.842^**^, *p* < 0.01), *AaANS* (*r* = 0.623^**^, *p* < 0.01), *AaLAR* (*r* = 0.904^**^, *p* < 0.01), and *AaANR* (*r* = 0.660^**^, *p* < 0.01) and correlated well with proanthocyanin accumulation (*r* = 0.754^**^, *p* < 0.01). This implies that AaMYB3 regulates the early biosynthetic genes in the flavonoid pathway (*AaCHS* and *AaF3H*) and the late biosynthetic genes *AaDFR* and *AaANS*, as well as the PA-specific genes *AaLAR* and *AaANR*. Similarly, the persimmon PA regulator DkMyb2 upregulated the expression of *DkCHS*, *DkDFR*, *DkANS*, *DkLAR*, and *DkANR* in transgenic persimmon calluses^56^. In contrast, the expression pattern of *AabHLH1* was similar to that of *AaF3′H* (*r* *=* 0.645^**^, *P* < 0.01) in the spathes of the two cultivars. A previous study of the expression of the flavonoid pathway genes suggested that *AaF3H* and *AaANS* are coregulated, while *AaDFR* and *AaF3′H* are separately regulated^23,26^. Our previous study suggested that AaMYB2 regulates the expression of *AaF3H* and *AaANS*, and possibly *AaCHS*, in the anthocyanin biosynthetic pathway of anthurium, while the expression pattern of *AaMYB2* differed dramatically from that of *AaDFR* and *AaF3′H*^29^. In this study, a gene expression analysis suggested that *AaMYB3* specifically coregulates *AaDFR*, *AaLAR*, and *AaANR* expression in the PA pathway, while AabHLH1 is probably involved in the regulation of *AaF3′H*. These results extend our understanding of the regulation of flavonoid biosynthesis in anthurium.

These results suggest that AaMYB3 functions as a negative regulator of anthocyanin accumulation and a positive regulator of PA accumulation in the anthurium spathe. This may occur by the upregulation of the expression of the genes for the enzymes specific to PAs biosynthesis, such as *AaLAR* and *AaANR*, and the acceleration of the reduction of leucocyanidin and anthocyanidin to promote the biosynthesis of PAs and repress the accumulation of anthocyanins. Based on the analyses described above, we propose a model of how anthocyanin and PA accumulation are affected by the anthocyanin regulator AaMYB2 and the PA regulator AaMYB3 in the anthurium spathe (Fig. 6c). In the red, pink, and purple spathes in which anthocyanin and PA accumulate simultaneously, both *AaMYB2* and *AaMYB3* are expressed, and the two TFs coregulate the expression of *AaCHS*, *AaF3H*, and *AaANS*. AaMYB3 specifically actives *AaDFR*, *AaLAR*, and *AaANR* expression. Therefore, anthocyanins and PAs accumulate together in the spathe. In the white and green spathes in which only PA accumulates, the expression of *AaMYB2* is negligible, and AaMYB3 activates the PA biosynthetic pathway, causing the accumulation of PAs. AabHLH1 interacts with AaMYB3 and AaMYB2 in the transcriptional regulation process.

## Electronic supplementary material

Supplementary data
