# Supplementary material for: AaMYB3 interacts with AabHLH1 to regulate proanthocyanidin accumulation in Anthurium andraeanum (Hort.)—another strategy to modulate pigmentation
Source: Hortic Res. 2019 Jan 1;6:14. doi: 10.1038/s41438-018-0102-6 (PMC6312548; doi:10.1038/s41438-018-0102-6)
Supplement: Supplementary file 1 — Supplementary data [file 41438_2018_102_MOESM1_ESM.docx]

**The AaMYB3 interacts with the AabHLH1 to regulate proanthocyanidin accumulation in *Anthurium andraeanum* (Hort.)——another strategy for pigmentation modulation**

**Running title: AaMYB3 and AabHLHl from Anthurium**

Chonghui Li^1,3^, Jian Qiu^2^, Surong Huang^1,3^, Guangsui Yang^1,3*^, Junmei Yin^1,3*^

CH. Li and J. Qiu contributed equally to this work

^1^ Tropical Crops Genetic Resources Institute, the Chinese Academy of Tropical Agricultural Sciences (CATAS) / Key Laboratory of Crop Gene Resources and Germplasm Enhancement in Southern China, Ministry of Agriculture, Danzhou 571737, China

^2^ Rubber Research Institute, CATAS/ Key Laboratory of Biology and Genetic Resources of Rubber Tree, Ministry of Agriculture, Danzhou 571737, China

^3^ The Engineering Technology Research Center of Tropical Ornamental Plant Germplasm Innovation and Utilization, Hainan Province, Danzhou 571737, China

^*^Corresponding author: Junmei Yin and Guangsui Yang

e-mail: [yinjunmei2011@sina.com](mailto:yinjunmei2011@sina.com) (J. Yin); [13976572870@163.com](mailto:13976572870@163.com) (G. Yang)

**Supplementary data**

Supplementary data are available online.

Supplementary Fig. S1.

Supplementary Fig. S2.

Supplementary Fig. S3.

Supplementary Table S1.

Supplementary Table S2.

**Supplementary Fig. S1**. Sequence alignment of AabHLH1 and the known anthocyanin bHLH regulators in other species. Identical nucleotides are shown on a black background, and gaps are indicated by dashes. MYB interaction region and bHLH domain are shown. ACT-like domain are indicated in the red box.

**Supplementary Fig. S2**. The relative expression analysis of genes in flavonoid biosynthesis pathway of based on relative expression in the transgenic *tt2* lines overexpressing *AaMYB3*. Color bar: Log_2_ (fold changes).

**Supplementary Fig. S3**. Analysis the role of *AaMYB3* and *AabHLH1* in anthurium. Detection for the content of total proanthocyanidin and anthocyanin as well as the expression levels of *AaMYB3*, *AabHLH1*, and flavonoid biosynthetic genes in the developmental spadix of cultivar ‘Vitara’ (**a**) and ‘Tropical’ (**b**). The data were presented as the mean ± SD (n = 3). Values with different letters are significantly different according to Duncan's multiple range tests at the 5% level.

**Supplementary Table S1** A list of q–PCR primers used in this study

| Genes | GenBank accession number | Forward Primer (5'-3') | Reverse Primer (5'-3') |
| --- | --- | --- | --- |
| *AaMYB3* | MH349476 | TGGAACAGCAGCCTCAGCAA | CTTGGTCCGGATAACCCCAT |
| *AabHLH1* | MH349477 | TCATCGTCCTACGGTCCCTG | CCATTTGCTTCATCTGGGACTC |
| *AaCHS* | DQ421809.1 | GCTGAGCGAGTACGGCAACA | TTCTCCTGCACGGGGAACTT |
| *AaCHI* | KU356776.1 | TCAGGGACTCTCACGATTGGG | TGCTGGCCGACGATGGACTC |
| *AaF3H* | AY232493.1 | CATGTCCGGGGGCAAGAA | CCATCAACCCCTCGCTGTAC |
| *AaF3'H* | KJ624416.1 | GCTGCCTTCCTCAAGACCCAT | AGGTGGACGGAGCAGAGCTT |
| *AaDFR* | AY232494.1 | CTCGCCCTCATCACAAGGAA | ACGTCGAACTCAGGGTAGCG |
| *AaANS* | AY232495.1 | CGTGGAGATCCTCAGCAACG | ACCTTGGGTCTTGCGGAAGA |
| *AaUFGT* | KC507547.1 | CTGCGTTCCTCAGGGCACTCG | GCCCCAGACTCCCGAAACAGA |
| *AaLAR* | MH349479 | TCGACCAGTTTCCCCTGATTG | TAGGTGTAGGGGATGTGGGCA |
| *AaANR* | MH349478 | GGTGAAGCGGGTGATCTTG | TTCTCTGTCATCGTCTTGGCA |
| *AaCYP* | JN602201.1 | GGCAAGCCCCTCCACTACAA | GCCCTGGCACATGAACTGGG |
| *AaUBQ5* | JN602199.1 | TCCGCATCCAGAAGTGGTAC | TGCCGTCGTGGATCTCGTAG |
| *Nt4CL* | U50845.1 | TCATTGACGAGGATGACGAG | TGGGATGGTTGAGAAGAAGG |
| *NtPAL* | X78269.1 | ATTGAGGTCATCCGTTCTGC | ACCGTGTAACGCCTTGTTTC |
| *NtCHS* | AF311783.1 | TTGTTCGAGCTTGTCTCTGC | AGCCCAGGAACATCTTTGAG |
| *NtCHI* | AB213651.1 | GTCAGGCCATTGAAAAGCTC | CTAATCGTCAATGCCCCAAC |
| *NtF3H* | AB289450.1 | CAAGGCATGTGTGGATATGG | TGTGTCGTTTCAGTCCAAGG |
| *NtF3'H* | KF856279.1 | CAAGGTGGCGTATGCTTAGGA | GTGGTGCACACGTTCAACAGTT |
| *NtFLS* | AB289451.1 | GAACTTGAAGGGAAAAGGGG | TCCCTGTAGGAGGGAGGATT |
| *NtDFR* | EF421429.1 | AACCAACAGTCAGGGGAATG | TTGGACATCGACAGTTCCAG |
| *NtANS* | AB723683.1 | AATGACCAACCCTCTGGCAAT | GGCCAGATGGATAAGTCGCA |
| *NtUFGT* | AB723685.1 | GAGTGCATTGGATGCCTTTT | CCAGCTCCATTAGGTCCTTG |
| *NtLAR* | AM827419.1 | TCAAGGTCCTTTACGCCATC | ACGAACCTGCTTCTCTTTGG |
| *NtANR1* |  | CATTTGACTTTCCCAAACGC | ATTGGGCTTTTGAGTTGTGC |
| *NtANR2* | AM791704.1 | TGTTCCCACTTGGGATGATA | TGCACCTATACTCTGTTAGTGGC |
| *NbActin* | AY179605.1 | AATGATCGGAATGGAAGCTG | TGGTACCACCACTGAGGACA |
| *NtL25* | L18908.1 | AGGCTGTCAAGTCAGGATCAAC | ATTGCAGACTCTGTGGTGAGG |
| *AtCHS* | DQ062416.1 | CGCATCACCAACAGTGAACAC | TCCTCCGTCAGATGCATGTG |
| *AtCHI* | NM_115370.4 | CCGGTTCATCGATCCTCTTC | ATCCCGGTTTCAGGGATACTATC |
| *AtF3H* | NM_001203121.1 | CAGATCGTTGAGGCTTGTGAGA | GACGAGTCATATCCGCCACTAAGT |
| *AtFLS* | NM_120951.3 | CCGTCGTCGATCTAAGCGAT | CGTCGGAATCCCGTGGT |
| *AtF3'H* | NM_120881.3 | GCTCTCGCCGGAGTATTCAA | CCAGCGACGCCTTGTAAATC |
| *AtDFR* | AK221622.1 | ACGTGGTTACTTTGTTCGTGCCA | CCGTTTATGGCATCATCGTAGC |
| *AtANS* | AK317414.1 | AAGAGCTCGAGAGCATCAACGA | GATGCCTTTTTCAGCTCCTCAA |
| *AtUFGT* | NM_102790.4 | TGGCAGTTCTCGCGTTTTTCC | CGACGCGTTTGATCTTGCGGTG |
| *AtANR* | DQ446384.1 | TTAGCTCGTGCCCATTTGTTT | ACAAGCCCTCTTCGAATTCTGA |
| *AtTT8* | AJ277509.1 | CGCATTGTCACCGGAGGATT | TTTCCTGGCATCCCAGATGGA |
| *AtTT2* | AJ299452.1 | CCGCAAAAGACTTCCCAAAA | GCACCTAATCGCCTTTGTACGT |
| *AtActin* | NM_105873.3 | AGGAAGTTGCTGCTCCTGTC | ACCTTCTCAGCTACAACAACCGTC |
| *AtUBQ1* | NM_115119.4 | TGAGCCTTCCTTGATGATGCT | GCACTTGCGGCAAATCATCT |

**Supplementary Table S2** Pearson’s correlation (*r*) of expression levels of *AaMYB3* and *AabHLH1* with anthocyanin and proanthocyanidin (PA) biosynthetic genes as well as total anthocyanin and PA content in the developing spathe and spadix of cultivars ‘Vitara’ and ‘Tropical’

| Cultivar | Tissue |  | Genes, TAC and TPC |  | *AaMYB3* |  | *AabHLH1* |
| --- | --- | --- | --- | --- | --- | --- | --- |
|  |  |  |  |  | *r* |  | *r* |
| ‘Vitara’ | Spathe |  | *AaCHS* |  | 0.868^**^ |  |  |
|  |  |  | *AaF3H* |  | 0.858^**^ |  |  |
|  |  |  | *AaF3′H* |  |  |  | 0.952^**^ |
|  |  |  | *AaDFR* |  | 0.936^**^ |  |  |
|  |  |  | *AaANS* |  | 0.894^**^ |  |  |
|  |  |  | *AaLAR* |  | 0.953^**^ |  |  |
|  |  |  | *AaANR* |  | 0.967^**^ |  |  |
|  |  |  | TAC |  | –0.741^**^ |  |  |
|  |  |  | TPC |  | 0.840^**^ |  |  |
|  |  |  |  |  |  |  |  |
|  | Spadix |  | *AaCHS* |  | 0.941^**^ |  |  |
|  |  |  | *AaCHI* |  | 0.618^*^ |  |  |
|  |  |  | *AaF3H* |  | 0.982^**^ |  |  |
|  |  |  | *AaDFR* |  | 0.823^**^ |  |  |
|  |  |  | *AaANS* |  | 0.911^**^ |  |  |
|  |  |  | *AaUFGT* |  |  |  | 0.954^**^ |
|  |  |  | *AaLAR* |  | 0.927^**^ |  |  |
|  |  |  | *AaANR* |  |  |  | 0.959^**^ |
|  |  |  |  |  |  |  |  |
| ‘Tropical’ | Spathe |  | *AaCHS* |  |  |  | 0.697^**^ |
|  |  |  | *AaCHI* |  |  |  |  |
|  |  |  | *AaF3H* |  |  |  | 0.528^*^ |
|  |  |  | *AaF3′H* |  |  |  | 0.983^**^ |
|  |  |  | *AaDFR* |  | 0.940^**^ |  |  |
|  |  |  | *AaANS* |  |  |  | 0.985^**^ |
|  |  |  | *AaUFGT* |  |  |  |  |
|  |  |  | *AaLAR* |  | 0.887^**^ |  |  |
|  |  |  | *AaANR* |  | 0.961^**^ |  |  |
|  |  |  |  |  |  |  |  |
|  | Spadix |  | *AaCHS* |  | 0.972^**^ |  |  |
|  |  |  | *AaCHI* |  |  |  | 0.777^**^ |
|  |  |  | *AaF3H* |  | 0.978^**^ |  |  |
|  |  |  | *AaF3′H* |  |  |  | 0.975^**^ |
|  |  |  | *AaDFR* |  | 0.962^**^ |  |  |
|  |  |  | *AaANS* |  | 0.590^*^ |  |  |
|  |  |  | *AaUFGT* |  |  |  | 0.991^**^ |
|  |  |  | *AaLAR* |  | 0.816^**^ |  |  |
|  |  |  | *AaANR* |  | 0.815^**^ |  |  |

^*^ Significant at *p* = 0.05, ^**^ significant at *p* = 0.01
